# A cross-sectional and longitudinal study between association of n-3 polyunsaturated fatty acids derived from fish consumption and high-density lipoprotein heterogeneity

**DOI:** 10.1007/s00380-017-1082-4

**Published:** 2017-11-20

**Authors:** Shigemasa Tani, Rei Matsuo, Kenji Kawauchi, Tsukasa Yagi, Wataru Atsumi, Atsushi Hirayama

**Affiliations:** 1 Department of Health Planning Center, Nihon University Hospital, 1-6 Kanda-Surugadai, Chiyoda-ku, Tokyo, 101-8309 Japan; 2 Department of Cardiology, Nihon University Hospital, Tokyo, Japan; 3 Division of Cardiology, Department of Medicine, Nihon University School of Medicine, Tokyo, Japan

**Keywords:** Apolipoprotein A-1, Docosahexaenoic acid, Eicosapentaenoic acid, High-density lipoprotein, n-3 Polyunsaturated fatty acid

## Abstract

Decreased high-density lipoprotein (HDL) particle size, cholesterol poor, apolipoprotein A-I-rich HDL particles leading to smaller HDL particle size, may be associated with an anti-atherosclerotic effect. The data are sparse regarding the relationship between n-3 polyunsaturated fatty acids [n-3 PUFAs: eicosapentaenoic acid (EPA), docosahexaenoic acid (DHA)] and HDL particle size. This study was designed as a hospital-based cross-sectional study to investigate the relationship between the serum levels of n-3 PUFAs and the HDL-cholesterol/apolipoprotein A-1 ratio, as estimated by the HDL particle size, in patients with the presence of one or more risk factors for atherosclerotic cardiovascular disease (ASCVD). Six hundred and forty sequential patients were enrolled in this study. The serum levels of EPA and DHA showed a strong correlation (*r* = 0.736, *p* < 0.0001). However, in a multivariate regression analysis after adjustment for ASCVD risk factors, increased serum DHA (*β* = − 0.745, *p* = 0.021), but not serum EPA (*β* = − 0.414, *p* = 0.139) or EPA + DHA (*β* = 0.330, *p* = 0.557) level, was identified as an independent indicator of decreased HDL particle size. In 476 patients followed up for at least 6 months, the absolute change (Δ) in the HDL-cholesterol/apolipoprotein A-1 ratio decreased significantly as the quartile of the Δ DHA level increased (*p* = 0.014), whereas no significant difference in the Δ HDL-cholesterol/apolipoprotein A-1 ratio was noted with the increase in the quartile of the Δ EPA level. Moreover, a multivariate regression analysis identified increased DHA level and decreased estimated low-density lipoprotein (LDL) particle size measured relative to the mobility value of LDL with polyacrylamide gel electrophoresis (i.e., relative LDL migration: LDL-Rm value), as independent predictors of decreased HDL-cholesterol/apolipoprotein A-1 ratio (*β* = − 0.171, *p* = 0.0003 and *β* = − 0.142, *p* = 0.002). The results suggest that increased serum DHA level, but not EPA level, might be associated with decreased HDL-cholesterol/apolipoprotein A-1 ratio, an indicator of estimated HDL particle size. Further studies are needed to investigate the useful clinical indices and outcomes of these patients.

*Clinical Trial Registration Information* UMIN (http://www.umin.ac.jp/), Study ID: UMIN000010603.

## Introduction

The Japanese people are one of the leading nations with high levels of fish consumption in the world [1], and it has been suggested that the cardiovascular protective effects of n-3 polyunsaturated fatty acids (n-3 PUFAs) such as eicosapentaenoic acid (EPA) and docosahexaenoic acid (DHA) derived from fish intake have bearing on the suppression of coronary artery disease (CAD) morbidity [2, 3].

Not only low-density lipoprotein (LDL) metabolism but high-density lipoprotein (HDL) metabolism as well is closely involved in the progress and suppression of arteriosclerosis [4]. We previously reported better responses of Japanese subjects to lipid lowering treatment in suppressing progress of coronary arteriosclerosis, compared with Europeans and Americans, and also described the significant involvement of improved HDL metabolism in those therapeutic responses [5]. Our recent study reports that a group of subjects showing high serum levels of EPA and DHA had low morbidity of CAD and high serum levels of apolipoprotein A-1 (apoA-1), a major functional carrier protein component of HDL particles [6]. However, there have been no studies to examine changes in HDL particle size that has a great bearing upon the cholesterol efflux capacity in the reverse cholesterol transport (RCT) system with a major functional role for the antiatheriosclerotic effect of HDL [7, 8]. Reports dealing with the relationship of n-3 PUFAs with HDL particle size are few as yet, and there is no unanimity of view in this regard [9–12].

In view of the inverse correlation between serum HDL-C level and CAD morbidity based on a number of epidemiological studies [13], the “HDL-C hypothesis” that an increase in serum HDL-C level results in attenuation of the risk of CAD has long been supported [14]. Nevertheless, large-scale clinical studies to examine the relation of serum HDL-C level to suppression of occurrence of CAD with the results disproving the hypothesis have followed one another [15–17]. It has been shown in recent years that the more pronounced is the HDL-cholesterol efflux capacity, the less conspicuous is the carotid intima-media thickness [18] and the less frequent are CAD events [19]. It is now recognized that, for prevention of atherosclerotic cardiovascular disease (ASCVD), it is important not so much to increase the serum HDL-C level as to improve the function of HDL [14].

It has been reported in recent years that small HDL particles with scanty lipid components and high protein contents (low HDL-C/apoA-1 ratio) are involved in the prevention of ASCVD [20–22] than large HDL particles.

We thus set up a hypothesis that the ASCVD-suppressive effect of n-3 PUFAs acquired through fish intake may have an association with the HDL particle size as an indicator of HDL function, or the low HDL-C/apoA-1 ratio. The aim of the present study was to examine the relationship of the HDL-C/apoA-1 ratio as a marker of HDL particle estimation and n-3 PUFAs (EPA, DHA) using cross-sectional and longitudinal study procedures.

## Method

### Study design and populations

This study was designed as a hospital-based cross-sectional and longitudinal studies to investigate the relationships between the serum HDL-C/apoA1 ratio as estimated the HDL particle size and serum n-3 PUFAs (EPA and DHA) levels in patients with one or more risk factors for ASCVD. Furthermore, we examined the relationship between the changes in the serum n-3 PUFAs level and the change in the serum HDL-C/apoA1 ratio in the cases that were available for additional measurements 6 months later. This study is a subanalysis of our previous study [6]. The study was conducted using a sample of 700 consecutive outpatients who had undergone regular examinations and blood examinations at the Cardiovascular Center of Nihon University Surugadai Hospital between June and October 2009.

The criterion for patient registration in the cross-sectional study was the presence of one or more risk factors for atherosclerosis. The diagnostic criteria for coronary risk factors were as follows: a diagnosis of hypertension was made when the systolic pressure was ≥ 140 mmHg, the diastolic pressure was ≥ 90 mmHg, or the patient was taking anti-hypertensive medication. Diabetes mellitus (DM) was defined as a fasting plasma glucose concentration ≥ 126 mg/dL and an HbA1c ≥ 6.5% or current treatment with anti-diabetic agents. A diagnosis of dyslipidemia was made when the LDL cholesterol (LDL-C) level was ≥ 140 mg/dL, triglyceride (TG) level was ≥ 150 mg/dL, the HDL-C level was ≤ 40 mg/dL, or if the patient was already taking lipid-lowering medication. A diagnosis of hyperuricemia was made when the serum uric acid level was ≥ 7.0 mg/dL or the patient was taking medications. The severity of chronic kidney disease (CKD) was determined based on the estimated glomerular filtration rate (eGFR) using the abbreviated Modification of Diet in Renal Disease (MDRD) Study equation, modified by a Japanese coefficient [23]; CKD was defined as eGFR < 60 mL/min/1.73 m^2^. Obesity was defined as a body mass index (BMI) ≥ 25 kg/m^2^.

Patients were not enrolled if they met any of the following exclusion criteria: hepatic dysfunction (alanine aminotransferase and aspartate aminotransferase ≥ 2 times the upper limit of the normal values), known malignant disease, refusal to provide consent for participation in the study, current treatment with n-3 PUFAs, or the diagnosis of acute coronary syndrome within 3 months prior to the study. The Nihon University Surugadai Hospital Ethics Committee approved the design and purpose of the study.

### Measurement of laboratory parameters

Fasting blood samples were collected early in the morning after a 12-h fast. The serum fatty acid levels were measured using capillary gas chromatography (SRL Co., Ltd., Tokyo, Japan). The serum total cholesterol (TC), HDL-C, and TG levels were measured using standard methods. LDL-C levels were estimated using the Friedewald formula [24]. The RLP-C level was measured using an immunoadsorption assay (SRL). The serum apo and Lp (a) levels were determined using turbidimetric latex agglutination assays (Daiichi Pure Chemicals Co., Ltd., Tokyo, Japan). The malondialdehyde-modified LDL (MDA-LDL) level was measured using an enzyme-linked immunosorbent assay (SRL). The high-sensitivity C-reactive protein (hs-CRP) level was measured using a nephelometric assay (Behring Diagnostic Marburg, Germany). The relative LDL migration (LDL-Rm value), an indicator of LDLparticle size, was measured relative to the mobility value of LDL by performing polyacrylamide gel electrophoresis with the LipoPhor system (JOKO, Tokyo, Japan). The LDL-Rm value was calculated as the distance between the VLDL peak and the LDL peak divided by the distance between the VLDL peak and the HDL peak. A decrease in the LDL-Rm value indicates an increase in the LDLparticle size [25, 26].

### Statistical analysis

Data were expressed as the mean ± standard deviation for continuous variables and as percentages for discrete variables. For variables with a significantly skewed distribution, the data were expressed as the interquartile range. In a subset analysis performed according to quartiles of the serum markers, we used an analysis of variance (ANOVA) followed by Bonferroni’s adjustment for covariates if differences had been detected in the serum markers. A multivariate regression analysis was used to assess determinants of the independent variables of the HDL-C/apoA-1 ratio, as the estimated HDL particle size. The HDL-C/apoA-1 ratio was considered as the dependent variable, and the independent variables included age, gender, ASCVD risk factors, and the serum EPA, DHA, and EPA + DHA levels. In 476 patients followed up for at least 6 months, univariate and multivariate regression analyses were also performed to identify independent variables associated with the absolute change (Δ) in HDL/apoA-1 level. A *p* value less than 0.05 was considered to indicate statistical significance. All the statistical analyses were performed using the SPSS software program (SPSS Inc., Chicago, IL, USA) for Windows (version 12.0.1).

## Results

### Patients

We excluded 60 subjects from the analysis because of missing laboratory data. Therefore, finally, 640 subjects were included in the analysis. The patient characteristics and laboratory profile are shown in Tables 1 and 2. The EPA level ranged from 6.2 to 373.5 μg/mL [median, interquartile range; 63.0 μg/mL (40.1/92.6)], and the DHA level ranged from 35.7 to 451.8 μg/mL [128.9 μg/mL (100.8/60.8)]. In addition, there were no cardiovascular events during the 6-month follow-up period in the 476 patients followed up for at least 6 months.

**Table 1:** Patient characteristics (*N* = 640)

Male/female, *n* (%)	448 (68)/212 (32)
Age (years)	62.4 ± 14.2
BMI (kg/m^2^)	24.0 ± 3.9
Hypertension, *n* (%)	462 (70)
Diabetes mellitus, *n* (%)	179 (27)
HbA1c (%)	5.97 ± 0.76
Current smoking, *n* (%)	94 (14)
Dyslipidemia, *n* (%)	408 (62)
eGFR (ml/min/1.73 m^2^)	70.7 ± 18.4
CKD ≥ stage 3, *n* (%)	173 (27)
Number of risk factors	2.5 ± 1.4
Coronary artery disease, *n* (%)	145 (22)
Concomitant drug, *n* (%)	
Anti-platelets	186 (28)
ACEs/ARBs	309 (47)
β blockers	140 (21)
Calcium channel blockers	333 (50)
Lipid-modifying drugs	324 (49)

*BMI* body mass index, *Hb* hemoglobin, *eGFR* estimated glomerular flow rate, *CKD* chronic kidney disease, *ACE-I* angiotensin converting enzyme inhibitor, *ARB* angiotensin receptor blocker

**Table 2:** Laboratory profile

Lipids	
TC (mg/dL)	195 ± 37
LDL-C (mg/dL)	110 ± 30
HDL-C (mg/dL)	58 ± 17
TG (mg/dL)^a^	115 (86/150)
Non-HDL-C (mg/dL)	137 ± 34
MDA-LDL (U/L)	109 ± 44
Lp (a) (mg/dL)^a^	11 (5/25)
RLP-C (mg/dL)^a^	5.3 (4.0/7.7)
Apolipoproteins(mg/dL)	
apoA-1	147 ± 30
apo B	90 ± 21
apo C-II	4.5 ± 1.9
apo C-III	10.0 ± 3.3
Inflammatory markers	
hs-CRP (mg/L)^a^	0.5 (0.3/1.2)
PUFAs (μg/mL)	
EPA^a^	63 (40/93)
DHA^a^	129 (101/161)
AA	163 ± 44
EPA/AA^a^	0.397 (0.246/0.608)

*TC* total cholesterol, *LDL* low-density lpoprotein, *HDL* high-density lipoprotein, *MDA* malondialdehyde-modified, *Lp* lipoprotein, *RLP* remnant-like particle, *apo* apolipoprotein, *hs-CRP* hypersensitive C-reactive protein, *PUFA* polyunsaturated fatty acids, *EPA* eicosapentaenoic acid, *DHA* docosahexaenoic acid, *AA* arachidonic acid

^a^Median; interquartile range in parentheses

### Multivariate regression analysis of the relationship between HDL heterogeneity and serum n-3 PUFA levels

**Fig. 1:**
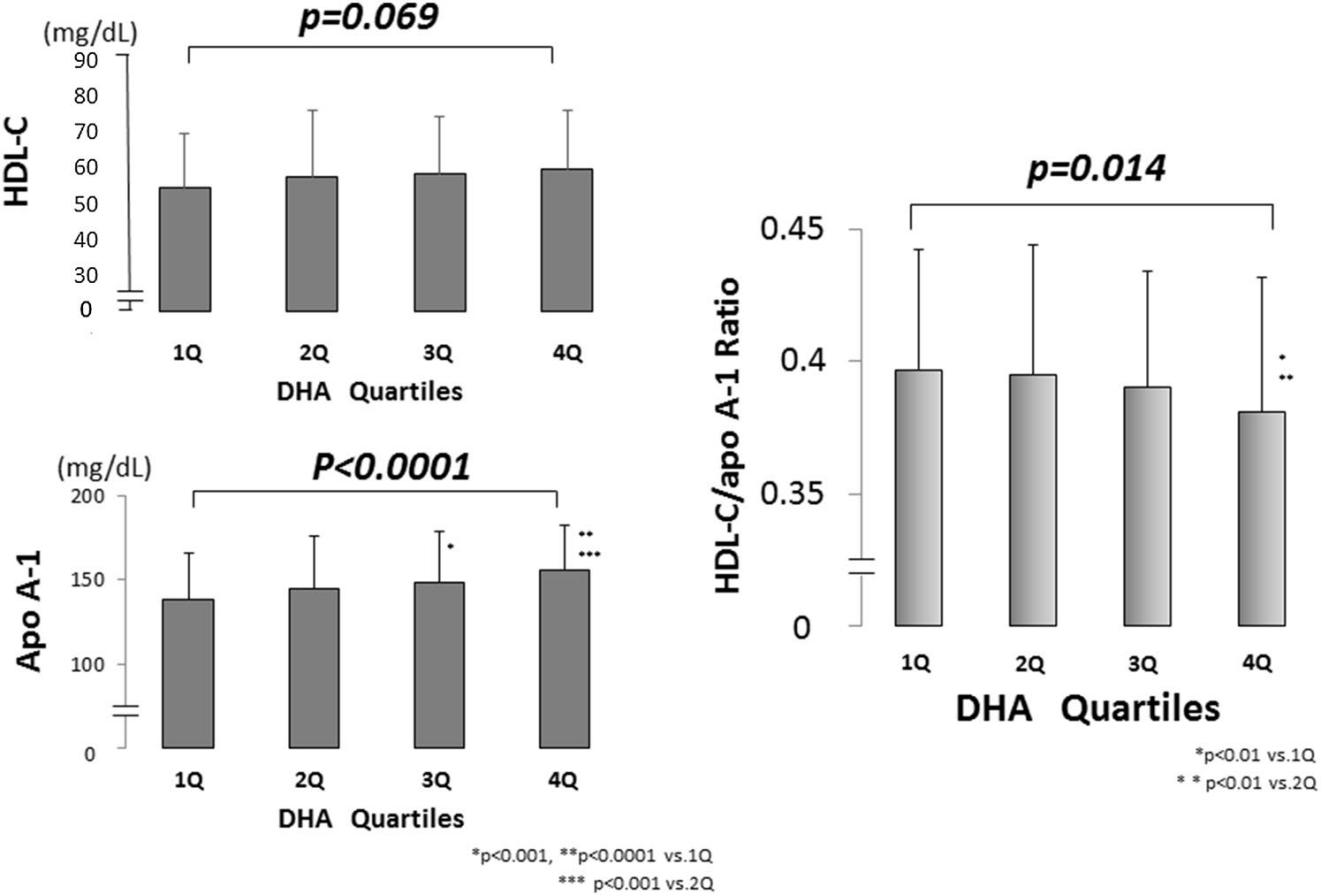
Association of the HDL-C/apoA-1 ratio with the serum DHA level. The patients were divided into quartiles of the DHA concentration. The DHA according to quartile was as follows: 35.7–100.7 μg/mL (quartile 1: *n* = 160), 100.8–128.7 μg/mL (quartile 2: *n* = 160), 128.9–160.5 μg/mL (quartile 3: *n* = 160), and 160.7–451.8 μg/mL (quartile 4: *n* = 160). The serum HDL-C level tended to increase as the serum DHA quartile increased, but it did not reach statistical significance. While the serum apoA-1 level increased significantly as the serum DHA quartile increased. The serum HDL-C/apoA-1 ratio accordingly decreased significantly as the serum DHA quartile increased. *HDL-C* high-density lipoprotein cholesterol, *apoA-1* apolipoprotein A-1, *DHA* docosahexaenoic acid

**Fig. 2:**
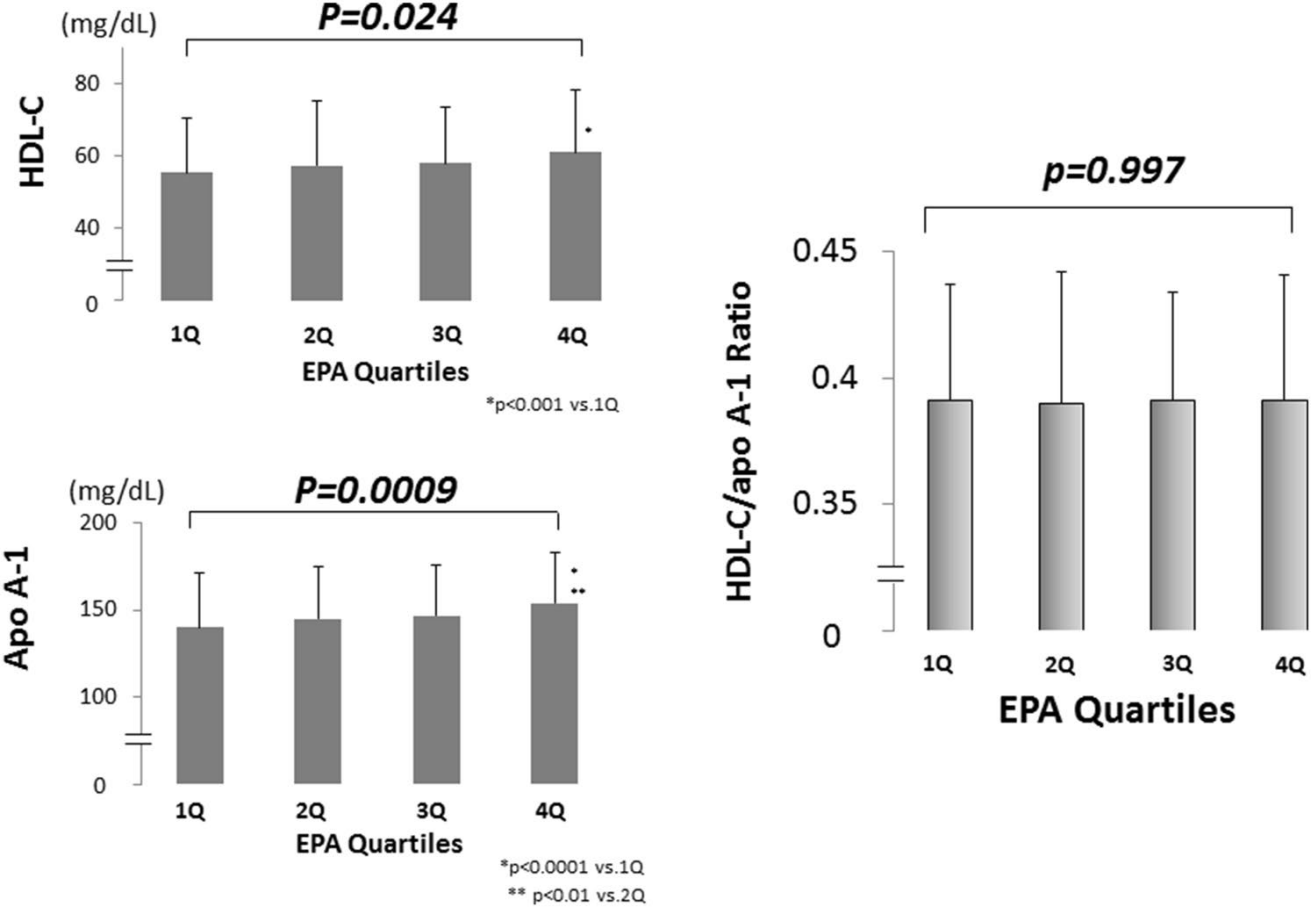
Association of the HDL-C/apoA-1 ratio with the serum EPA level. The patients were divided into quartiles of the EPA concentration. The ranges according to the quartile of EPA were follows: 6.2–40.0 μg/mL (quartile 1: *n* = 160), 40.1–62.8 μg/mL (quartile 2: *n* = 160), 63.0–92.2 μg/mL (quartile 3: *n* = 160), and 92.4–373.5 μg/mL (quartile 4: *n* = 160). The serum HDL-C level increased significantly as the serum EPA quartile increased, while the serum apoA-1 level also increased significantly as the serum EPA quartile increased. Accordingly, no significant change in the serum HDL-C/apoA-1 ratio is found as the serum EPA quartile increased. *HDL-C* high-density lipoprotein cholesterol, *apoA-1* apolipoprotein A-1, *EPA* eicosapentaenoic acid

The serum levels of EPA and DHA showed a strong correlation (r = 0.736, *p* < 0.0001). However, in the multivariate analysis after adjustment for ASCVD risk factors, serum DHA, but not serum EPA or EPA + DHA, was identified as an independent indicator of HDL particle size (Table 3). Associations between HDL particle size and serum n-3 PUFAs levels are shown in Figs. 1 and 2.

**Table 3:** Multivariate regression analysis of the relationship between HDL-heterogeneity and the serum n-3 PUFA levels (*N* = 640)

Independent variable	*β*	*p* value
Male gender	− 0.262	< 0.001
Age	− 0.117	0.003
BMI	− 0.248	< 0.001
Hypertension	− 0.082	0.026
Diabetes mellitus	− 0.037	0.297
Dyslipidemia	− 0.162	0.0005
Current smoking	− 0.072	0.039
Lipid-modifying drugs	− 0.002	0.961
EPA^a^	0.414	0.139
DHA^a^	− 0.745	0.021
EPA + DHA^a^	0.330	0.557

The abbreviations are the same as in Tables 1 and 2, *β* = standard partial regression coefficient, multiple *R* = 0.566; *F* = 25.744; *p* < 0.0001

^a^ Log-transformed value was used

### Comparison of the absolute changes of the serum HDL-C/apoA-1 ratio according to the quartiles with the absolute change (Δ) in serum DHA and EPA levels

**Fig. 3:**
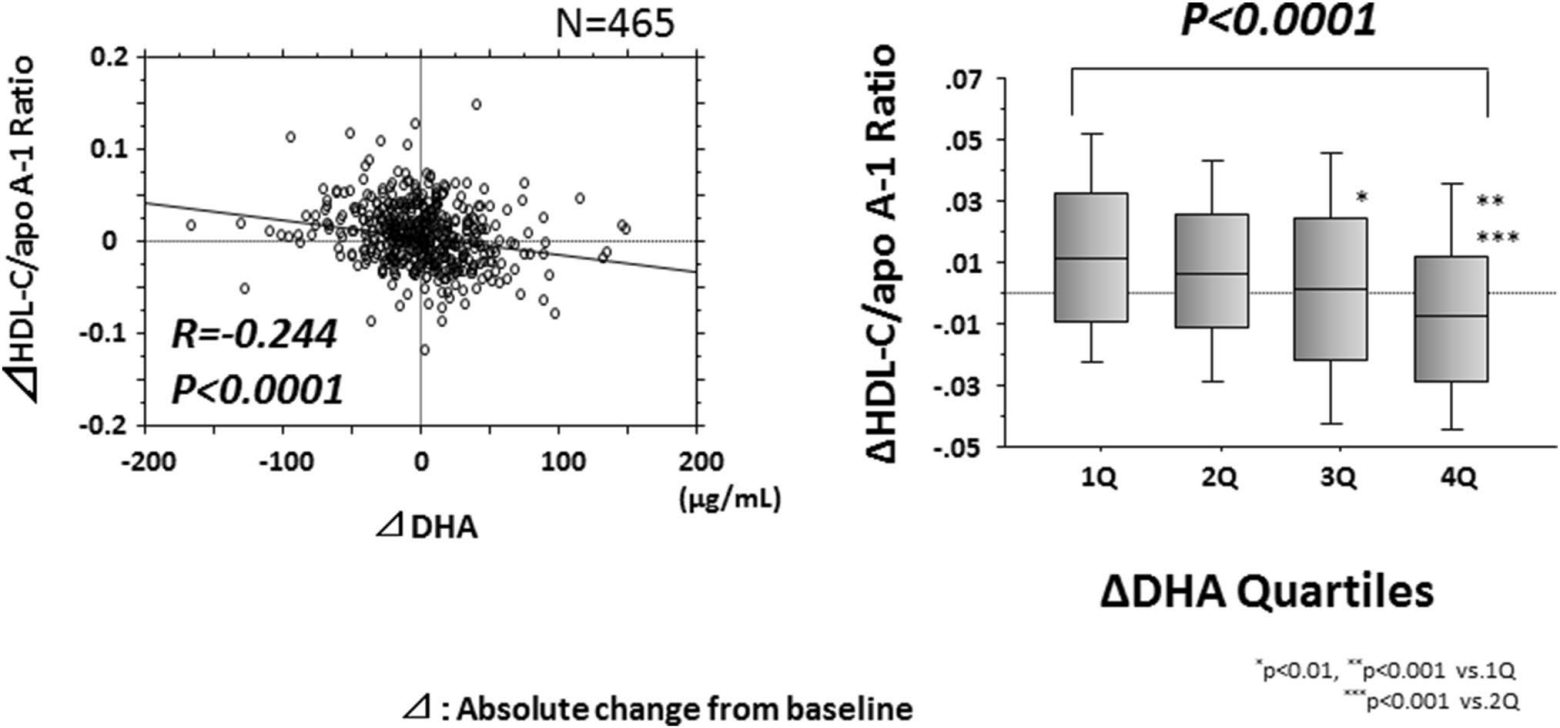
Association of the Δ HDL-C/apoA-1 ratio with the Δ serum DHA level. The Δ HDL-C/apoA-1 ratio correlated negatively with the Δ DHA level. Similarly, the Δ HDL-C/apoA-1 ratio decreased significantly as the Δ DHA quartile increased. *HDL-C* high-density lipoprotein cholesterol, *apoA-1* = apolipoprotein A-1, *DHA* docosahexaenoic acid, *Δ* absolute change from baseline to 6-month follow-up

Among the subjects of this cross-sectional study, we investigated the association between the absolute change (Δ) in DHA quartile and the Δ serum HDL-C/apoA-1 ratio in 476 patients who could be followed up for at least 6 months. The Δ serum DHA level ranged from − 163.5 to 151.0 ng/dL (mean ± SD 2.6 ± 37.7 ng/dL). The patients were divided into quartiles according to the Δ serum DHA level, as follows: quartile 1, − 163.5 to − 17.5 ng/dL (*n* = 115), quartile 2, − 17.4 to 2.8 ng/dL (*n* = 117), quartile 3, 2.9 to 20.9 ng/dL (*n* = 114), and quartile 4, 21.0 to 151.0 ng/dL (*n* = 116). The Δ serum HDL-C/apoA-1 level increased significantly as the quartile of the Δ serum DHA level increased (Fig. 3), whereas no significant difference in the Δ HDL-C/apoA-1 ratio was noted with increase in the quartile of the Δ serum EPA level (data not shown).

### Univariate and multivariate regression analyses to identify factors correlated with the absolute change in the serum HDL-C/apoA-1 ratio

In this cross-sectional study, we confirmed that increased serum DHA level was an important factor which causes a decrease in HDL particle size. Therefore, we investigated the relationship between the absolute changes (∆) in the serum HDL level and the ∆ serum HDL-C/apoA-1 ratio in order to examine their causal relationships, using in a longitudinal method. To investigate the effects of the increase and decrease of the serum DHA level on the Δ serum HDL-C/apoA-1 ratio, we carried out univariate and multivariate regression analyses using the Δ serum HDL-C/apoA-1 ratio as a dependent variable and the Δ serum DHA level as an independent variable, with adjustments for gender, age, ASCVD risk factors, and the Δ LDL-migration index (LDL-Rm value), as indicators of LDL-particle size, in 476 patients who could be followed up at least 6 months after this cross-sectional study. These analyses revealed that an increased serum DHA level and LDL-Rm value are independent predictors of a decrease in the serum HDL-C/apoA-1 ratio (Table 4).

**Table 4:** Univariate and multivariate regression analysis to identify factors correlated with the absolute change in the serum HDL-C/apoA-1 ratio, (*N* = 476)

Univariate			Multivariate		
Variable	*r*	*p* value	*β*	*t*	*p* value
∆ DHA	− 0.205	< 0.0001	− 0.171	− 3.679	0.0003
∆ LDL-Rm value	− 0.139	0.005	− 0.142	− 3.059	0.0023
Age	0.036	0.429			
Male gender	− 0.062	0.177			
BMI	0.046	0.312			
Smoking	0.037	0.424			
Hypertension	0.180	0.727			
Diabetes mellitus	− 0.049	0.280			
Dyslipidemia	− 0.048	0.299			
Lipid-modifying drugs	0.001	0.985			

All variables correlated with the ∆ HDL-C/apoA-1 ratio at *p* < 0.05 in the univariate regression analysis were entered into the multivariate model. The abbreviations are the same as in Tables 1 and 2, LDL-Rm = relative LDL migration. A decrease in the LDL-Rm value indicates an increase in the LDL particle size [25, 26]. *r* = correlation coefficient; *β* = standard partial regression coefficient, multiple *R* = 0.244, *F* = 14.460, *p* < 0.0001

### Association of the HDL-C/apoA-1 ratio and LDL-Rm value with the serum TG level

**Fig. 4:**
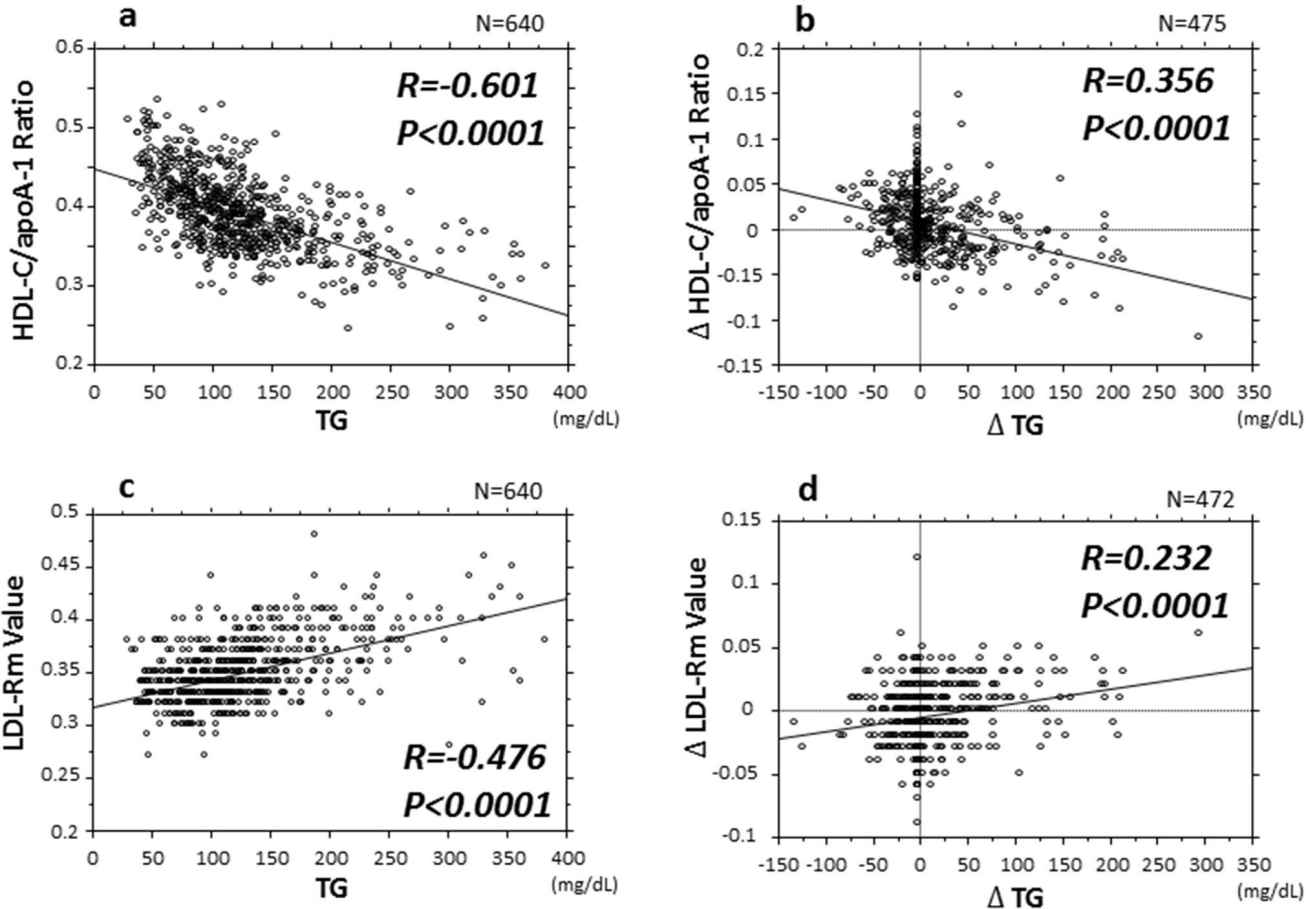
Title: Association between the HDL-C/apoA-1 ratio and LDL-Rm value associated, an indicator of LDL particle size with the serum TG level. *HDL-C* high-density lipoprotein cholesterol, *apoA-1* apolipoprotein A-1, *LDL-Rm* relative LDL migration, *TG* triglyceride, *Δ* absolute change from baseline to 6-month follow-up

Next, we investigated the relationship between HDL, LDL particle size, and the serum TG level which greatly affects the HDL- and LDL metabolism. The serum TG level was negatively correlated with the HDL-C/apoA-1 ratio and positively correlated with the LDL-Rm value (Fig. 4a, c). Furthermore, in the 476 patients followed up for at least 6 months, the ∆ TG level showed a similar negative correlation with the ∆ HDL-C/apoA-1 ratio and a similar positive correlation with the ∆ LDL-Rm value (Fig. 4b, d).

## Discussion

In this study, we have shown the following points. An elevated serum DHA concentration was found to be an independent prognostic factor for decrease in the HDL-C/apoA-1 ratio as an estimate of the HDL particle size whereas an increase in the serum EPA concentration did not prove to be a prognostic factor. These findings suggest that HDL in a group of subjects showing high serum DHA levels represents smaller particles.

Furthermore, an additional study with a longitudinal method revealed that elevated DHA levels were an independent predictor of decreased HDL particle size. Due to its observational design, this study was unable to establish a causal relationship between the results, but the combination of the results of the 2 studies with different (cross-sectional and longitudinal) designs strongly suggests that an increase in the serum DHA level was associated with a decrease of the HDL particle size in patients with one or more risk factors for ASCVD.

No unanimity of opinion has been achieved in reports dealing with the relationship between n-3 PUFAs and HDL particle size (intervention studies with administration of n-3 PUFAs, or association with serum n-3 PUFAs concentration) [9–12], or association between HDL particle size and development of CAD [27–30]. This may be attributed to disparities in the characteristics of study subjects (e.g., healthy subjects, a history of CAD) or use of different assay methods for evaluating HDL particle size. Superko et al. reported in their review article that the lack of unanimity of views on the results of clinical studies examining n-3 PUFAs and their ASCVD-suppressing effects came from differences in baseline serum n-3 PUFAs levels and dose of n-3 PUFAs in the groups of study subjects [31].

Density gradient ultracentrifugation, nondenaturing gradient gel electrophoresis, and nuclear magnetic resonance spectroscopy are the methods that are usually employed to measure HDL particle size, but they are difficult to apply in clinical settings due to their cost and complexity [8]. As one apoA-1 molecule is bound to each single HDL particle, however, the HDL-C/apoA-1 ratio has been reported to serve as a simple indicator of HDL particle size [32]. Both small HDL particles prior to cholesterol efflux and post-cholesterol efflux large HDL particles occur within arteriosclerotic plaque where the RCT system is activated. In recent years, there have been reports purporting to underline the importance of small HDL particles with strong cholesterol efflux capacity as an indicator of activation of the RCT system [7]. Due to the effect that lipid-poor HDL particles have in facilitating a reverse cholesterol transport system, we believe that cholesterol poor, apo A-I-rich HDL particles (i.e., producing a lower HDL-C/apoA-I ratio) are likely to reduce the risk of ASCVD. Similar studies, which have shown that the quality of HDL matters more than quantity, support this hypothesis [4].

The patients enrolled in the present study were those with a high risk of ASCVD possessing risk factors for arteriosclerosis. Sung and co-workers who conducted studies using the HDL-C/apoA-1 ratio as an indicator of HDL particle size, as in our study, reported that the lower the HDL-C/apoA-1 ratio, the lower was the mortality of CVD and coronary artery calcium [20, 21]. Meanwhile, a study investigating development of CKD among healthy subjects yielded negative results indicating that the lower the HDL-C/apoA-1 ratio, the higher was the morbidity of CKD [33]. As described earlier, the disparity of results depending on subjects enrolled in studies may be relevant to the degree of progression of arteriosclerosis due to lipid metabolism abnormality.

Not only the metabolism of LDL, which is predominantly involved in the progression and suppression of atherosclerosis, but also that of HDL, is greatly affected by the serum TG level [34]. As can be seen from Fig. 4a, HDL particles are prone to downsizing when a subject is in a hypertriglyceridemic state. Fournier et al. also reported a negative correlation between the HDL-C/apoA-1 ratio and the serum TG level, which lends support to our results [35]. It has been described that HDL particles are downsized in such a state, and that downsized HDL particles show depressed function, i.e., they are dysfunctional HDL particles [34]. However, it has also been reported that TG-rich, apoA-1-poor small dense HDL has an anti-atherogenic effect in patients with metabolic syndrome [36]. These points represent issues that still need to be clarified. We, therefore, examined the changes in the HDL particle size caused by variations of the serum TG level using a longitudinal research technique (Fig. 4b). The study demonstrated that the lower the serum TG level and the greater the likelihood of improvement of the hypertriglyceridemia, the greater the tendency for the HDL particles to grow into cholesterol-rich particles of larger size. This phenomenon may reflect conversion of small HDL particles into larger-sized HDL particles upon activation of RCT [7].

The present study also showed that under a hypertriglyceridemic state, the LDL particle size diminishes to yield increasingly downsized LDL particles [34] (Fig. 4c). This phenomenon, as for the case of changes of the HDL particle size, also may indicate, as shown in Fig. 4d, that improvement of hypertriglyceridemia results in amelioration of LDL metabolism, with a consequent trend toward increase of the LDL particle size, leading to suppression of the effect of LDL in promoting atherosclerosis progression.

Although it was a longitudinal study with temporal elements taken into account rather than an intervention study involving the use of lipid metabolism-improving drugs or lifestyle improvement, the present study was no more than an observational study; therefore, no reasonable conclusions can be drawn as to causal relationship of the findings gained. Nevertheless, the findings may show the interrelation between HDL and LDL metabolism.

**Fig. 5:**
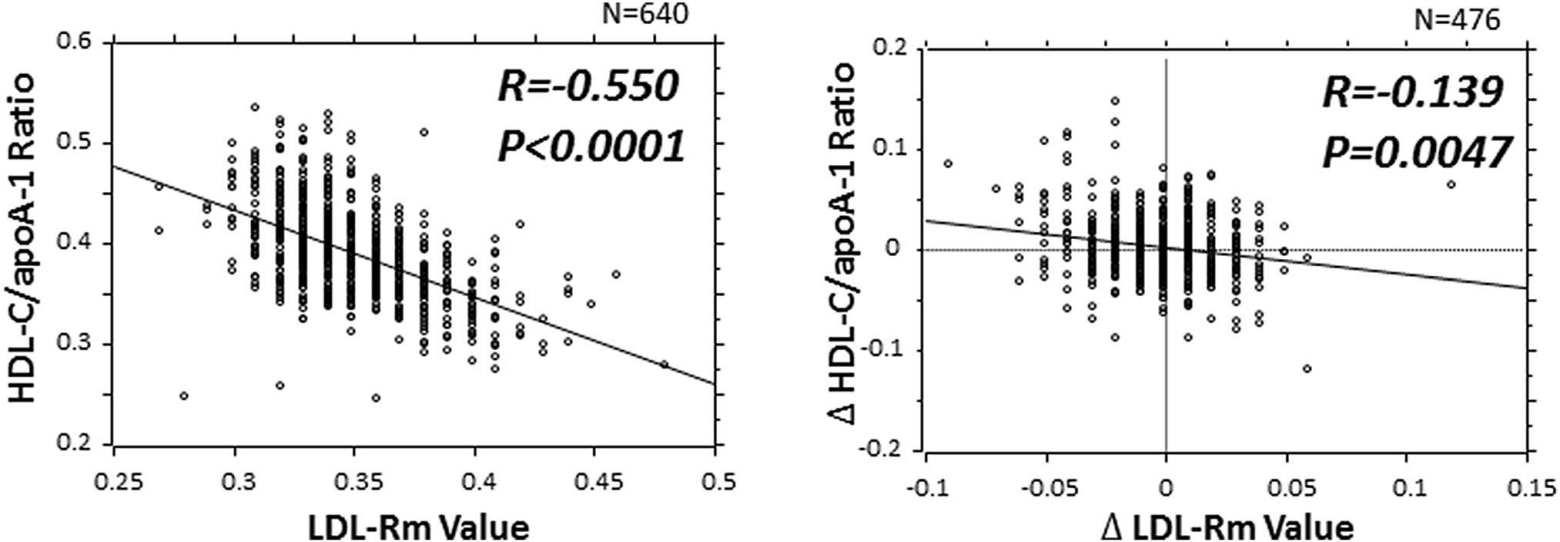
Association between HDL-C/apoA-1 ratio AND LDL-Rm value. A positive correlation was observed between the LDL-Rm value and HDL-C/apoA-1, and the ∆ LDL-Rm value was also found to be weakly correlated with the ∆ HDL-C/apoA-1 ratio. *HDL-C* high-density lipoprotein cholesterol, *apoA-1* apolipoprotein A-1, *LDL-Rm* relative LDL migration, *Δ* absolute change from baseline to 6-month follow-up

In conjunction with the above phenomenon, the present study shows the possibility that downsizing of LDL particles potently giving rise to progression of arteriosclerosis as well as elevation of serum DHA concentration is a determining factor for downsizing of HDL particles. It would seem probable that the RCT system may be activated contrarily as a result of increased small HDL particles under the circumstances where progressive downsizing of LDL particles is associated with the consequent facilitation of arteriosclerosis progression due to the presence of risk factors for atherosclerosis. Likewise, increased levels of lecithin-cholesterol acyltransferase activity have been reported as an RCT-activating factor under the circumstances of progressing arteriosclerosis in the presence of lipid metabolism abnormality [37, 38]. Based on the above presumptions, we prepared Fig. 5 by adopting the changes in the LDL particle size and changes in the HDL particle size as indicators of RCT activation.

It has been reported that n-3 PUFAs derived from fish intake are endowed with a variety of protective effects on the cardiovascular system [3]. Our present research focuses on HDL metabolism which is deeply involved in suppression of atherosclerotic progression. A recent study in vitro has demonstrated an HDL-cholesterol efflux capacity-activating effect of EPA [39]. Although it might appear inappropriate for us to refer unitarily to disparities in respective study results regarding n-3 PUFAs and the RCT system because of the differences in research procedures, it would seem to be reasonable to infer that n-3 PUFAs play some role in improving HDL function. We are hopeful that our present results will lay some foundation for elucidation of the mechanism whereby fish intake provides a cardiovascular event-suppressing effect.

## Study limitations and clinical implications

Firstly, as several methods are available for evaluation of HDL particle size, investigations with other evaluation methods have to be pursued [8]. Secondly, another concern lies in the lack of biological testing of the hypothesis on which this study is based, i.e., small HDL particles = high cholesterol countertransport activity and large HDL particles = low cholesterol countertransport activity. Thirdly, no investigation has been done as to HDL particle downsizing and HDL function under a hypertriglyceridemic state. Intervention studies with DHA formulations are needed to elucidate the underlying mechanisms. Fourthly, investigation of the phospholipids, which account for the largest part of the constituents of HDL particles and which are partially involved in the function of HDL, is to be pursued for precise evaluation of the effects of HDL particle size [7, 40]. HDL particles were only evaluated in terms of the serum HDL-C level and apoA-1 level in this study, which did not include investigation of the HDL particle constituents. Various approaches have been attempted to determine the functions of HDL [8], and it is considered that evaluation of the function of HDL using a combination of approaches is necessary to examine the suppressive effect of HDL on ASCVD. Finally, this study does not demonstrate the relationship between the HDL-C/apoA-1 ratio, serum DHA level, and clinical indices or/and outcomes. In the future, an interventional study to investigate the causal relationship is needed.

## Conclusion

Although there are numerous unresolved issues with regard to the differences in the ASCV-protective effects between EPA and DHA, an increased serum DHA level may be associated with decreased HDL-C/apoA-1 ratio, an indicator of estimated HDL particle size. Further studies are needed to investigate the useful clinical indices and outcomes of these patients.
